# Serum concentrations of small dense low‐density lipoprotein cholesterol and lipoprotein(a) are related to coronary arteriostenosis in Takayasu arteritis

**DOI:** 10.1002/jcla.23966

**Published:** 2021-10-28

**Authors:** Si Chen, Haixia Luan, Jianxun He, Yan Wang, Shuang Liu, Yongzhe Li, Xiaoli Zeng, Hui Yuan

**Affiliations:** ^1^ Department of Clinical Laboratory Beijing Anzhen Hospital Capital Medical University Beijing China; ^2^ Department of Clinical Laboratory Peking Union Medical College Hospital Peking Union Medical College Chinese Academy of Medical Science Beijing China

**Keywords:** coronary arteriostenosis, lipoprotein(a), small dense low‐density lipoprotein cholesterol, Takayasu arteritis

## Abstract

**Background:**

Serum small dense low‐density lipoprotein cholesterol (sdLDL‐C) and lipoprotein(a) [Lp(a)] levels are related to coronary disease, but their specific associations with coronary arteriostenosis in Takayasu arteritis (TA) have not been ascertained. This study explored the correlations between serum sdLDL‐C and Lp(a) levels and coronary arteriostenosis in TA patients as well as the degree of artery stenosis.

**Methods:**

This retrospective study included 190 TA patients and 154 healthy subjects. TA patients were divided into three categories based on the degree of coronary stenosis: Group I, stenosis >50%; Group II, stenosis 1%–50%; and Group III, stenosis 0%. Independent risk factors for coronary arteriostenosis in TA were identified by logistic regression, followed by receiver operating characteristic curve analysis to determine the specificity and sensitivity of risk factors and Youden's Index score calculation to determine the cutoff points.

**Results:**

Takayasu arteritis patients had significantly higher serum levels of sdLDL‐C and Lp(a) than healthy controls (*p* < 0.0001). The total cholesterol, triglyceride, LDL‐C, sdLDL‐C, and Lp(a) levels and the sdLDL‐C/LDL‐C ratio in Group I were significantly higher than those in Groups II and III (*p* < 0.05). An elevated serum sdLDL‐C level elevated the risk of coronary arteriostenosis by 5‐fold (cutoff value, 0.605 mmol/l). An increased serum Lp(a) level increased the risk of coronary arteriostenosis by 3.9‐fold (cutoff value, 0.045 g/l). An elevated sdLDL‐C/LDL‐C ratio increased the risk of coronary arteriostenosis by 2.1‐fold (cutoff value, 0.258).

**Conclusions:**

Serum sdLDL‐C and Lp(a) levels and sdLDL‐C/LDL‐C ratio may be used as diagnostic factors for coronary arteriostenosis in TA patients.

## Graphical abstract

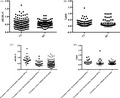

## 1 INTRODUCTION

Takayasu arteritis (TA) is a rare vasculitis that mainly involves the aorta and its major branches.^1^ TA is mainly seen in young women aged 20–40 years and is most often encountered in Japan, Southeast Asia, India, and Mexico.^2^ Froving and Loken first reported coronary arteriostenosis in TA in 1951.^3^ Since then, the incidence of coronary arteriostenosis in TA has varied in literature reports, depending on the study location and cohort. For example, some studies have reported that 10%–30% of TA patients had coronary arteriostenosis,^4^, ^5^, ^6^, ^7^, ^8^, ^9^, ^10^, ^11^ and another study reported 7.7%.^12^ However, research has consistently shown that coronary arteriostenosis is a risk factor for adverse outcomes and increased mortality in TA patients.^13^, ^14^, ^15^ Due to nonspecific clinical features, TA patients with coronary arteriostenosis might be misdiagnosed or inappropriately treated.^12^ As a result, detection of coronary stenosis in TA patients principally depends on computed tomography angiography (CTA); however, CTA can only detect 53.2% coronary stenosis in TA patients.^16^ Therefore, it is imperative to develop methods for accurate and timely diagnosis of TA patients with coronary arteriostenosis to facilitate appropriate clinical management. The identification of blood biomarkers for TA with coronary arteriostenosis has been an active area of research in the field.

Dyslipidemia plays a role in the pathogenesis of TA, and the lipid profiles in TA patients are pro‐atherogenic.^17^, ^18^ A previous study has shown that abnormal lipid metabolism, an elevated ratio of apolipoprotein B (apoB)/apolipoprotein A1 (apoA1), and reduced levels of apoA1 and high‐density lipoprotein cholesterol (HDL‐C) are associated with higher disease activity in TA,^17^ and these results were confirmed in other studies.^19^, ^20^, ^21^, ^22^ These findings support the premise that abnormal lipid profiling is closely linked to the development of TA.

Small dense low‐density lipoprotein cholesterol (sdLDL‐C) is small and highly dense relative to other LDL‐C particles and thought to be more atherogenic than the large buoyant LDL (lb‐LDL) due to greater penetration into the arterial wall, a prolonged plasma half‐life, lower hepatic LDL receptor affinity, and higher susceptibility to oxidation.^23^, ^24^ sdLDL‐C is independently correlated with the progression of atherosclerosis in the Chinese population.^25^, ^26^, ^27^, ^28^ However, whether the serum sdLDL‐C level is correlated with TA involving the coronary arteries is not clear. Lipoprotein(a) (Lp(a)), including the major components LDL‐C, apoA, and apoB,^29^ has long been considered an independent risk factor for coronary disease.^30^ Although one case report showed a significantly elevated serum level of Lp(a) in a TA patient,^31^ no systemic study has been performed to examine the link between serum levels of Lp(a) and TA involving the coronary arteries. This study aimed to explore the relationship between serum sdLDL‐C and Lp(a) levels and the disease activity of TA patients, and whether elevated serum sdLDL‐C and Lp(a) concentrations can be used as diagnostic factors in Chinese TA patients.

## 2 MATERIALS AND METHODS

### 2.1 Patient selection

One hundred ninety TA patients who were diagnosed with TA based on the criteria defined by the 1990 American College of Rheumatology (ACR) and were treated between September 2015 and August 2019 at Beijing Anzhen Hospital were registered in this retrospective study.^32^ Additionally, 154 healthy controls were screened during their physical examinations and enrolled in this study. If TA patients had other autoimmune disorders, they were excluded from the subsequent study. Based on the Numano criteria, TA was divided into six subtypes (I, IIa, IIb, III, IV, and V) by catheterography or CTA,^33^, ^34^ TA disease activity was assessed according to the criteria recommended by Kerr et al.^35^ The patients with TA underwent CTA examination first, and radiologists determined whether patients had coronary stenosis. If the results of CTA were abnormal, patients need to undergo coronary angiography to decide the degree of stenosis. According to the findings of coronary angiography, the patients were divided into three categories: Group I, coronary stenosis >50%; Group II, coronary stenosis 1%–50%; and Group III, coronary stenosis (0%). Therefore, Groups I and II had coronary arteriostenosis. All participants signed an informed consent form. This study was authorized by the Ethics Committee of Beijing Anzhen Hospital, Capital Medical University.

### 2.2 Laboratory measurement

Early in the morning, venous blood samples were obtained from fasting TA patients and healthy controls. Our team previously compared methods for measuring sdLDL‐C,^36^ and a Beckman AU5400 (US) automatic biochemical analyzer was used in the present study to measure novel and traditional lipid parameters and biochemical indicators, including the sdLDL‐C, Lp(a), total cholesterol (TC), LDL‐C, HDL‐C, and triglyceride (TG) concentrations within 5–6 h after sample collection. The Sysmex XE‐2100 was used to determine complete blood counts. The experiments were accomplished following the manufacturer's instructions. In addition, all measurements were analyzed using the continuous monitoring method, and appropriate quality control was carried out before these analyses.

### 2.3 Statistical analysis of data

The statistical analyses were performed using SPSS 23.0 (SPSS Inc.). The measurement data were tested for normality. The normal distribution data are presented as mean ± standard error of the mean (SEM) and were compared with an independent sample t‐test. Data with a skewed distribution are presented as median and interquartile range (25%, 75%Q) and were contrasted with the Mann–Whitney‐Wilcoxon test. The chi‐square test or Fisher's exact test was used to compare with counting data. One‐way analysis of variance (ANOVA) or the Kruskal‐Wallis test followed by Dunn's post hoc test was used to compare differences in variables among three or more groups. When a *p*‐value was <0.05, the Mann‐Whitney‐Wilcoxon test or unpaired Student's *t*‐test was used to perform multiple comparisons between two groups. Logistic regression was used to determine independent risk factors for TA patients with coronary arteriostenosis. Receiver operating characteristic (ROC) curves were used to determine the specificity and sensitivity of novel and traditional risk factors for coronary stenosis >50% and coronary arteriostenosis. The cutoff values of diagnostic indicators were determined by Youden's Index scores (sensitivity+specificity‐1). A *p*‐value <0.05 was regarded as statistically meaningful.

## 3 RESULTS

### 3.1 Clinical features of participants

In the present study, 190 TA patients (women, 93.7%; mean age, 36.03 ± 12.70 years) and 154 healthy controls (women, 88.3%; mean age, 38.03 ± 9.07 years) were enrolled. In TA patients, the three most common clinical symptoms were malaise (74.2%), headache (50.0%), and chest distress (28.6%), and Numano V type was the most common subtype. Also, 47 TA patients had active disease according to the NIH criteria. Detailed demographic and baseline clinical characteristics of these participants are given in Table 1.

**TABLE 1:** Clinical characteristics of TA patients

	No. of TA patients, *n* (%)
Constitutional symptoms	
Fever	8 (4.21)
Malaise	135 (71.05)
Arthralgia/arthritis	12 (6.32)
Headache	91 (47.89)
Chest distress/pain	52 (27.37)
Carotidynia	16 (8.42)
Active^a^	47 (24.74)
Vascular findings	
Claudication	15 (7.89)
Bruits	127 (66.84)
Weakened pulse	143 (75.26)
Pulse deficit	58 (30.53)
Asymmetric BP	104 (54.74)
Hypertension	90 (47.37)
Coronary artery involvement (stenosis >50%)	21 (11.05)
Coronary artery involvement (stenosis 1–50%)	19 (10.00)
Numano subtypes	
I	9 (4.74)
IIa	4 (2.11)
IIb	36 (18.95)
III	7 (3.68)
IV	17 (8.95)
V	117 (61.58)

Abbreviations: BP, blood pressure; TA, Takayasu arteritis.

Italic values are statistically significant.

^a^ According to the National Institutes of Health (NIH) criteria.

### 3.2 Comparison of clinical manifestations and outcomes between active and inactive TA patients

**FIGURE 1:**
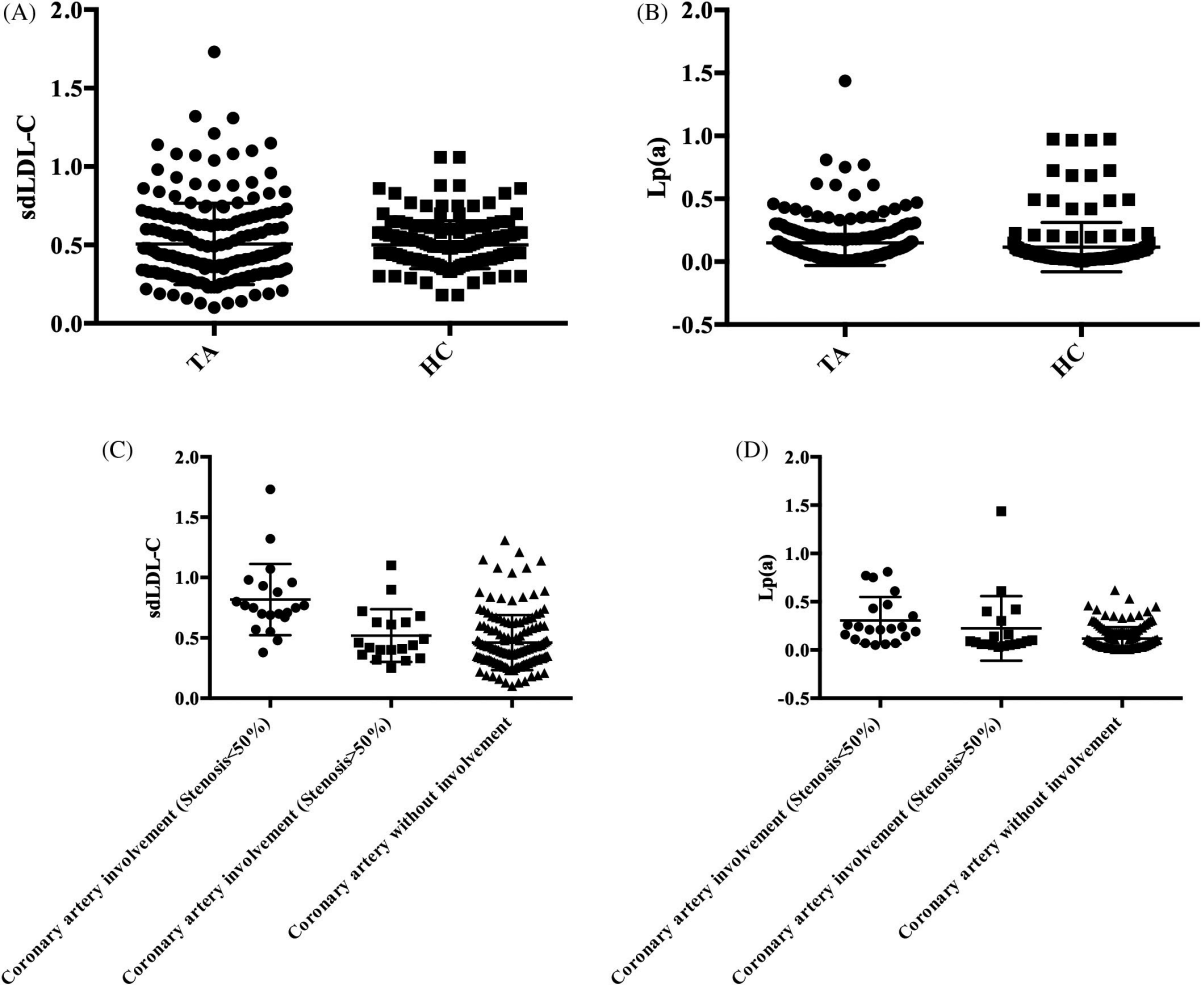
Comparison of serum concentrations of sdLDL‐C and Lp(a) among the different groups. A, Serum sdLDL‐C concentrations were significantly different between TA patients and healthy subjects; B: serum Lp(a) concentrations were significantly different between TA patients and healthy subjects (*p *< 0.0001); C: Group II and III patients had lower sdLDL‐C concentrations than Group I patients (*p *< 0.05); D: Group I patients had a higher Lp(a) concentration than Group II and III patients (*p* < 0.05). TA, Takayasu arteritis; Group I: coronary stenosis >50% in TA patients; Group II: coronary stenosis 1%–50% in TA patients; and Group III: coronary stenosis (0%) in TA patients

As shown in Figure 1, TA patients had higher TC, TG, LDL‐C, Lp(a), and sdLDL‐C concentrations and a significantly higher sdLDL‐C/LDL‐C ratio than the healthy controls. TA patients with active disease had a higher incidence of claudication than those with inactive disease (*p* = 0.018). Four TA patients with active disease had Numano subtype IIa, whereas no cases of Numano IIa were found in TA patients with inactive disease (*p* = 0.003). Also, patients with active TA had significantly lower serum HDL‐C levels (*p* < 0.0001) but higher LDL‐C levels (*p* = 0.037) than those with inactive TA. However, there were no significant differences in the serum sdLDL‐C and Lp(a) levels between patients with active and inactive TA. The clinical details of active and inactive TA patients are presented in Table 2.

**TABLE 2:** Demographic and clinical characteristics and laboratory findings of TA patients with active and inactive disease

	HC (*n* = 154)	TA (*n* = 190)	*p*‐value	Active (*n* = 47)	Inactive (*n* = 143)	*p*‐value
Female	136 (88.31%)	178 (93.68%)	.12	44 (93.62%)	134 (93.71%)	.983
Age (years)	38.03 ± 9.07	36.03 ± 12.70	.620	35.79 ± 12.06	36.11 ± 12.94	.972
Constitutional symptoms						
Fever	–	8 (4.21%)	–	3 (6.38%)	5 (3.50%)	.663
Malaise	–	135 (71.05%)	–	34 (72.34%)	101 (70.63%)	.822
Arthralgia/arthritis	–	12 (6.32%)	–	2 (4.26%)	10 (6.99%)	.746
Headache	–	91 (47.89%)	–	20 (42.55%)	71 (49.65%)	.398
Chest distress/pain	–	52 (27.37%)	–	14 (29.79%)	38 (26.57%)	.668
Carotidynia	–	16 (8.42%)	–	3 (6.38%)	13 (9.09%)	.782
Vascular findings						
Claudication	–	15 (7.89%)	–	8 (17.02%)	7 (4.90%)	. ***018***
Bruits	–	127 (66.84%)	–	30 (63.83%)	97 (67.83%)	.613
Pulsation weakened	–	143 (75.26%)	–	34 (72.34%)	109 (76.22%)	.592
Pulse deficit	–	58 (30.53%)	–	11 (23.40%)	47 (32.87%)	.222
Asymmetric BP	–	104 (54.74%)	–	23 (48.94%)	81 (56.64%)	.357
Hypertension	–	90 (47.37%)	–	27 (57.45%)	63 (44.06%)	.111
Laboratory data						
TC	3.16 (2.79–3.59)	3.92 (3.32–4.49)	***<.0001***	3.96 (3.32–4.55)	3.91 (3.31–4.47)	.898
TG	0.90 (0.63–1.13)	1.17 (0.72–1.36)	***<.0001***	1.29 (0.69–1.63)	1.13 (0.73–1.31)	.540
HDL‐C	1.43 (3.81–4.56)	1.38 (1.13–1.58)	.052	1.20 (1.01–1.38)	1.44 (1.19–1.65)	***0.0001***
LDL‐C	1.48 (1.20–1.72)	2.11 (1.59–2.52)	***<.0001***	2.29±0.80	2.05±0.67	. ***037***
sdLDL‐C	0.50 (0.40–0.58)	0.51 (0.32–0.64)	.084	0.57 (0.34–0.72)	0.49 (0.32–0.59)	.103
Lp(a)	0.12 (0.02–0.09)	0.15 (0.03–0.19)	***<.0001***	0.16 (0.05–0.23)	0.15 (0.03–0.19)	.245
sdLDL‐C/LDL‐C	0.35 (0.27–0.42)	0.24 (0.19–0.26)	***<.0001***	0.24 (0.18–0.27)	0.23 (0.19–0.26)	.965
WBC	5.59 (4.26–6.30)	7.24 (4.91–8.40)	***<.0001***	7.40 (5.68–8.11)	7.19 (4.71–8.50)	.406
Hb	133.99 (125.00–138.00)	123.30 (114.75–133.00)	***<.0001***	120.45±14.41	124.24±14.67	.088
PLT	257.17 (216.00–300.00)	238.02 (193.75–278.25)	. ***004***	264.55±80.61	229.30±60.49	. ***011***
ALT	14.40 (9.00–19.00)	21.39 (11.00–24.00)	. ***002***	19.57 (9.00–20.00)	21.99 (11.00–26.00)	.135
Scr	59.88 (52.50–68.20)	54.62 (46.75–61.28)	***<.0001***	54.51 (44.70–63.30)	54.66 (47.20–60.70)	.966
ESR	5.58 (3.00–8.00)	10.00 (3.00–12.00)	. ***001***	19.66 (10.00–23.00)	6.80 (2.00–9.00)	***<.0001***
hs‐CRP	0.90 (0.37–1.03)	3.74 (0.13–2.29)	.283	9.49 (1.15–14.88)	1.85 (0.08–1.17)	***<.0001***
Numano subtypes						
I	–	9 (4.74%)	–	3 (6.38%)	6 (4.20%)	.829
IIa	–	4 (2.11%)	–	4 (8.51%)	0 (0.00%)	. ***003***
IIb	–	36 (18.95%)	–	6 (12.77)	30 (20.98%)	.213
III	–	7 (3.68%)	–	3 (6.38%)	4 (2.80%)	.493
IV	–	17 (8.95%)	–	3 (6.38%)	14 (9.78%)	.678
V	–	117 (61.58%)	–	28 (59.58%)	89 (62.24%)	.745

Note

Abbreviations: ALT, alanine aminotransferase; BP, blood pressure; ESR, erythrocyte sedimentation rate; Hb, hemoglobulin; HC, healthy controls; HDL‐C, high‐density lipoprotein cholesterol; hs‐CRP, hypersensitive C‐reactive protein; LDL‐C, low‐density lipoprotein cholesterol; Lp(a), lipoprotein(a) ; PLT, platelet count; Scr, serum creatinine; sd‐LDL‐C, small dense low‐density lipoprotein cholesterol; TA, Takayasu arteritis; TC, total cholesterol; TG, triglycerides; WBC, white blood cell count.

Italic values are statistically significant.

### 3.3 Comparison of clinical manifestations and outcomes between TA patients with and without coronary arteriostenosis

TA patients were assigned to three groups based on the degree of coronary stenosis: Group I (*n* = 21), at least one site of coronary stenosis >50%; Group II (*n* = 19), at least one site of coronary stenosis 1–50%; and Group III (*n* = 150), no coronary stenosis (0%). The onset age of Groups II and III patients was lower than that of Group I patients (*p* < 0.05). Numano subtype V was mainly observed in the Group I patients (76.19%). There were significant differences in the frequencies of systemic symptoms among patients of Group I, Group II, and Group III (*p* < 0.05), including arthralgia/arthritis, headache, and chest tightness/pain. For results related to vascular reaction, there were significant differences in the frequencies of pulse deficit, asymmetric blood pressure, and hypertension in the Group I, Group II and Group III (*p* < 0.05) patients. Concerning the lipid parameters, Group I had higher TC, TG, LDL‐C, sdLDL‐C, and Lp(a) levels and a higher sdLDL‐C/LDL‐C ratio compared with Groups II and III (*p* < 0.05, Table 3 and Figure 1). The variables that showed significant differences between the three groups (Table 3) were used in the multiple logistic regression models.

**TABLE 3:** Demographic and clinical characteristics and laboratory findings in TA patients with coronary artery involvement

	Group I (*n *= 21)	Group II (*n* = 19)	Group III (*n* = 150)	*p*‐value
Female	18 (85.71%)	19 (100.00%)	9 (6.00%)	.188
Age (years)	50.57 ± 11.66^a^ ^,^ ^b^	42.63 ± 9.62^b^	33.16 ± 11.46^c^	***<.0001***
Constitutional symptoms				
Fever	0 (0.00%)	2 (10.53%)	6 (4.00%)	.291
Malaise	18 (85.71%)	17 (89.47%)	101 (67.33%)	.045
Arthralgia/arthritis	0 (0.00%)^a^	7 (36.84%)^b^	5 (3.33%)	***<.0001***
Headache	2 (9.52%)^a^ ^,^ ^b^	9 (47.37%)	80 (53.33%)	. ***001***
Chest distress/pain	21 (100.00%)^a^ ^,^ ^b^	6 (31.58%)	25 (16.67%)	***<.0001***
Carotidynia	0 (0.00%)	0 (0.00%)	16 (10.67%)	.136
Vascular findings				
Claudication	3 (14.29%)	1 (5.26%)	11 (7.33%)	.551
Bruits	15 (71.43%)	16 (84.21%)	96 (64.00%)	.200
Pulsation weakened	15 (71.43%)	16 (84.21%)	112 (74.67%)	.572
Pulse deficit	13 (61.90%)^a^ ^,^ ^b^	0 (0.00%)^b^	45 (30.00%)	***<.0001***
Asymmetric BP	18 (85.71%)^b^	12 (63.16%)	74 (49.33%)	. ***004***
Hypertension	19 (90.48%)^a^	9 (47.37%)	62 (41.33%)	***<.0001***
Laboratory data				
TC	4.30 (3.94–4.70)^a^ ^,^ ^b^	3.87 (3.30–4.18)	3.87 (3.26–4.35)^d^	. ***016***
TG	2.11 (1.23–2.16)^a^ ^,^ ^b^	1.09 (0.74–1.32)	1.05 (0.68–1.23)^d^	***<.0001***
HDL‐C	1.23 (1.02–1.43)^b^	1.32 (1.15–1.63)	1.41 (1.17–1.60)	.099
LDL‐C	2.33 (1.97–2.61)^b^	2.11 (1.49–2.47)	2.07 (1.59–2.44)	.073
sdLDL‐C	0.82 (0.68–0.95)^a^ ^,^ ^b^	0.52 (0.36–0.63)	0.46 (0.31–0.58)^d^	***<.0001***
Lp(a)	0.31 (0.13–0.45)^a^ ^,^ ^b^	0.22 (0.06–0.30)	0.12 (0.03–0.18)^d^	***<.0001***
sdLDL‐C/LDL‐C	0.35 (0.30–0.38)^a^ ^,^ ^b^	0.25 (0.22–0.27)^c^	0.22 (0.18–0.24)^d^	***<.0001***
WBC	6.18 (4.51–6.97)	6.31 (4.15–8.01)	7.51 (5.08–8.63)	.152
Hb	119.57 (100.00–136.00)	124.37(119.00–133.00)	123.69 (115.00–132.25)	.364
PLT	208.05 (141.50–281.50)	256.00(159.00–345.00)	239.94 (201.00–276.00)	.214
ALT	31.90 (20.00–39.00)^a^ ^,^ ^b^	33.21 (12.00–31.00)	18.42 (10.00–21.00)^d^	***<.0001***
Scr	57.66 (44.95–64.95)	53.55 (47.40–58.30)	54.33 (46.83–61.05)	.917
ESR	10.43 (5.00–12.50)	11.00 (3.00–20.00)	9.78 (2.25–11.00)	.612
hs‐CRP	1.47 (0.16–1.98)	5.39 (0.11–8.65)	3.82 (0.11–2.79)	.294
Numano subtypes				
I	0 (0.00%)	0 (0.00%)	9 (6.00%)	.553
IIa	0 (0.00%)	2 (10.53%)	2 (1.33%)	.066
IIb	5 (23.81%)	3 (15.79%)	28 (18.67%)	.795
III	0 (0.00%)	1 (5.26%)	6 (4.00%)	.625
IV	0 (0.00%)	0 (0.00%)	17 (11.33%)	.087
V	16 (76.19%)	13 (68.42%)	88 (58.67%)	.250

Abbreviations: ALT, alanine aminotransferase; BP, blood pressure; ESR, erythrocyte sedimentation rate; Group I, TA patients with a site of coronary artery stenosis >50%; Group II, TA patients with a site of coronary artery stenosis <50%; Group III, TA patients without coronary artery stenosis; Hb, hemoglobulin; HDL‐C, high‐density lipoprotein cholesterol; hs‐CRP, hypersensitive C‐reactive protein; LDL‐C, low‐density lipoprotein cholesterol; Lp(a):lipoprotein(a); PLT, platelet count; Scr, serum creatinine; sd‐LDL‐C, small dense low‐density lipoprotein cholesterol; TA, Takayasu arteritis; TC, total cholesterol; TG, triglycerides; WBC: white blood cell count.

Italic values are statistically significant.

^a^ The difference between Group I and Group II is statistically significant.

^b^ The difference between Group I and Group III is statistically significant.

^c^ The difference between Group II and Group III is statistically significant.

^d^ The difference between patients with stenosis (Groups I and II) and those without (Group III) is statistically significant.

Multiple logistic regression analyses showed that sdLDL‐C (standardized β = 3.285, *p* = 0.005, OR = 5.706), Lp(a) (standardized β = 2.699, *p* = 0.019, OR = 3.858), sdLDL‐C/LDL‐C (standardized β = 3.769, *p* < 0.0001, OR = 2.115), and age (standardized β = 0.140, *p* < 0.0001, odds ratio [OR] = 1.151) were independent risk factors for coronary arteriostenosis in TA after adjustment for TC and TG levels (Table 4). The ROC curve analysis results for risk factors for coronary arteriostenosis in TA are given in Table 5.

**TABLE 4:** Determination of independent risk factors of coronary artery involvement by multivariate linear regression analysis

	Standardized β	Standard error	Wald χ^2^	95% confidence interval	OR	*p*‐value
Age	0.140	0.035	15.720	1.074–1.233	1.151	***<.0001***
TC	−0.628	0.410	2.348	0.325–1.757	0.534	.125
TG	0.146	0.326	0.201	0.125–2.015	1.708	.654
sdLDL‐C	3.285	1.460	5.064	0.215–3.020	5.706	. ***005***
Lp(a)	2.699	1.338	4.068	0.731–3.991	3.858	. ***019***
sdLDL‐C/LDL‐C	3.769	2.981	1.438	1.271–3.344	2.115	***<.0001***

Note

Abbreviations: Lp(a), lipoprotein(a); OR, odds ratio; sd‐LDL‐C, small dense low‐density lipoprotein cholesterol; TC, total cholesterol; TG, triglycerides.

Italic values are statistically significant.

**TABLE 5:** Sensitivity and specificity of risk factors for coronary artery involvement of TA

	Cutoff point	Youden's Index	AUC	Sensitivity (%)	Specificity (%)	95% CI	*p*‐value
sdLDL‐C	0.605	0.393	0.739	60.0	79.3	0.655–0.827	<.0001
Lp(a)	0.045	0.323	0.700	95.0	37.3	0.614–0.786	<.0001
sdLDL‐C/LDL‐C	0.258	0.560	0.821	70.0	86.0	0.751–0.890	<.0001
TC	3.595	0.228	0.601	77.5	45.3	0.510–0.693	.049
TG	1.035	0.375	0.727	77.5	40.0	0.643–0.812	<.0001
LDL‐C	2.175	0.188	0.574	57.5	61.3	0.478–0.671	.149

Note

Abbreviations: AUC, area under the receiver operating characteristic (ROC) curve; CI, confidence interval; LDL‐C, low‐density lipoprotein cholesterol; Lp(a), lipoprotein(a); sd‐LDL‐C, small dense low‐density lipoprotein cholesterol; TC, total cholesterol; TG, triglycerides.

### 3.4 Determination of cutoff values for sdLDL‐C and Lp(a) for predicting coronary arteriostenosis in TA

A serum cutoff point of 0.605 mmol/l for sdLDL‐C maximized the diagnostic efficacy in assessing coronary arteriostenosis in TA, with a sensitivity of 60.00% and a specificity of 79.30% (Table 5). A serum cutoff value of 0.045 g/L for Lp(a) maximized the diagnostic efficacy in assessing coronary arteriostenosis in TA, with a sensitivity of 95.00% and a specificity of 37.30% (Table 5). Table 6 shows the ROC curve analysis results for risk parameters for coronary stenosis >50% in TA. A serum cutoff point of 0.665 mmol/l for sdLDL‐C maximized the diagnostic efficacy in assessing coronary stenosis >50%, with a sensitivity of 81.00% and a specificity of 84.00% (Table 6). A serum cutoff point of 0.185 g/L for Lp(a) maximized the diagnostic efficacy in assessing coronary stenosis >50%, with a sensitivity of 66.70% and a specificity of 78.10% (Table 6).

**TABLE 6:** Sensitivity and specificity of risk factors for coronary stenosis (>50%) in TA patients

	Cutoff point	Youden's Index	AUC	Sensitivity (%)	Specificity (%)	95% CI	*p*‐value
sdLDL‐C	0.665	0.650	0.863	81.0	84.0	0.796–0.930	<.0001
Lp(a)	0.185	0.448	0.777	66.7	78.1	0.682–0.872	<.0001
sdLDL‐C/LDL‐C	0.273	0.745	0.916	85.7	88.8	0.868–0.963	<.0001
TC	4.41	0.394	0.693	61.9	77.5	0.597–0.790	.004
TG	1.215	0.597	0.853	85.7	74.0	0.784–0.922	<.0001
LDL‐C	2.385	0.353	0.653	61.9	73.4	0.552–0.754	.022

Note

Abbreviations: AUC, area under the receiver operating characteristic (ROC) curve; CI, confidence interval; LDL‐C, low‐density lipoprotein cholesterol; Lp(a), lipoprotein(a); sd‐LDL‐C, small dense low‐density lipoprotein cholesterol; TC, total cholesterol; TG, triglycerides.

## 4 DISCUSSION

To the best of our knowledge, this was the first study to investigate the correlations between serum sdLDL‐C and Lp(a) levels and coronary arteriostenosis in Chinese TA patients. We found TA patients had higher concentrations of serum sdLDL‐C and Lp(a) than healthy controls. After grouping patients by the degree of coronary stenosis, Group I had significantly higher levels of sdLDL‐C and Lp(a) than Groups II and III. We further identified that serum sdLDL‐C and Lp(a) levels and sdLDL‐C/LDL‐C ratio may be used as diagnostic factors for coronary arteriostenosis in TA patients.

Takayasu arteritis is a rare autoimmune inflammatory vasculitis. The prolongation of the process of inflammatory hyperplasia of the arterial intima and contraction of the fibrous matrix and the adventitia of the ascending aorta together cause coronary stenosis in TA patients.^37^ Acceleration of atherosclerosis caused by vasculitis may be another mechanism behind coronary arteriostenosis in TA.^38^ The rate of coronary arteriostenosis in TA is relatively low, and several studies have reported an incidence of coronary arteriostenosis in TA between 7% and 30%,^4^, ^5^, ^6^, ^7^, ^8^, ^9^ rates which are consistent with the incidence of ~21% in the present study. The prognosis of TA patients with coronary arteriostenosis is often poor, and many patients die of cardiac events.^39^ Hence, it is necessary to make an early diagnosis in TA patients, especially in the prestenotic phase, and coronary revascularization and bypass grafting should be undertaken promptly during the stable stage for TA patients.^40^, ^41^ Previously, inflammatory factors such as the erythrocyte sedimentation rate (ESR) and C‐reactive protein (CRP) level have been used to evaluate the activity of TA.^42^ Indeed, in the present study, we also detected significant differences in the ESR and CRP levels between active and inactive TA patients. However, no specific serum marker that can identify coronary arteriostenosis in patients with TA has been previously reported.

According to its size and density, sdLDL‐C, a subclass of LDL‐C, has a greater tendency for endothelial infiltration and is associated with a higher susceptibility to atherogenesis.^43^, ^44^ sdLDL‐C has a lower affinity for LDL receptors, resulting in a longer period of particle retention in circulation and increased sensitivity to glycosylation, oxidation, and scavenger receptor uptake.^44^ In addition, sdLDL‐C is more susceptible to oxidation and proteoglycan binding, consequently resulting in increased arterial thickness.^45^ Therefore, sdLDL‐C has a higher atherogenic profile. Relevant studies have shown that the determination of sdLDL‐C is helpful in risk assessment and the determination of cardiovascular residual risk.^46^ Recent studies have shown that some diseases including obesity,^44^, ^45^ metabolic syndrome (MetS),^45^, ^47^, ^48^ systemic hypertension,^49^ and hepatic diseases^50^, ^51^ are associated with high sdLDL‐C concentrations. Moreover, the sdLDL‐C/LDL‐C ratio has been used as an alternative risk indicator in some comorbidities associated with the cardiovascular system.^45^, ^49^, ^50^, ^51^, ^52^, ^53^, ^54^ In line with the previous findings, in the present study, we found that TA patients had higher sdLDL‐C levels than the healthy subjects and that the Group I TA patients had a higher sdLDL‐C level than the patients in Groups II and III, supporting the notion that serum sdLDL‐C levels are correlated with the severity of coronary stenosis in TA patients.

Lp(a), a LDL‐like lipid fraction, is a highly pro‐atherogenic lipid fraction and believed to contribute to the pathogenesis of atherosclerosis by carrying oxidized phospholipid (OxPL).^55^ Several studies have suggested a strong association between increased Lp(a) and cardiovascular disease, including stroke, and aortic stenosis.^56^ Also, Lp(a) was shown to be positively correlated with interleukin (IL)‐6 and CRP after acute myocardial infarcts,^57^, ^58^ indicating its involvement in immune and pro‐inflammatory responses. Similar to sdLDL‐C, in the present study, we also found that TA patients had a higher Lp(a) level than the healthy participants and that Group I had higher Lp(a) levels than Groups II and III, indicating that serum Lp(a) levels are associated with TA severity.

Abnormal blood lipid levels occur in many autoimmune diseases and are involved in all stages of the inflammatory process and the pathophysiology of different chronic autoimmune diseases such as systemic lupus erythematosus (SLE), rheumatoid arthritis (RA), and multiple sclerosis.^59^ Moreover, dyslipidemia plays a key role in the formation of atherosclerosis in SLE patients.^60^, ^61^, ^62^ Abnormal lipid metabolism is also present in TA patients^17^, ^19^, ^20^, ^21^, ^22^; however, no studies have evaluated the associations of serum sdLDL‐C and Lp(a) levels with disease activity and coronary arteriostenosis until now. In the present study, although we did not find a close correlation between serum sdLDL‐C and Lp(a) levels and TA activity, our results showed that an elevated serum level of sdLDL‐C increased the risk of coronary arteriostenosis in TA by 5.7‐fold. The cutoff values for the serum sdLDL‐C level for indicating coronary stenosis (>50%) were 0.605 and 0.665 mmol/l, respectively, with appreciable specificity and sensitivity levels. Similarly, an elevated Lp(a) level increased the risk of coronary arteriostenosis in TA by 3.9‐fold, with cutoff values for coronary stenosis (>50%) of 0.045 and 0.185 g/L, respectively, and appreciable specificity and sensitivity. In addition, an elevated sdLDL‐C/LDL‐C ratio increased the risk of coronary arteriostenosis in TA by 2.1‐fold, with cutoff values for coronary stenosis (>50%) of 0.258 and 0.273, respectively, and appreciable specificity and sensitivity. Therefore, evaluating the threshold of serum sdLDL‐C and Lp(a) levels and sdLDL‐C/LDL‐C ratio in clinical practice may enable clinicians to assess the risk of coronary arteriostenosis in TA patients as well as the degree of coronary stenosis, thus guiding appropriate clinical management of these patients.

The limitations of this study should be noted. First, this was a single‐center, retrospective study, which had some intrinsic shortcomings including sampling bias. Also, we did not take into consideration the potential effects of the treatments on serum levels of sdLDL‐C and Lp(a). Thus, our conclusions should be further corroborated in the future by multi‐center, prospective clinical studies.

In conclusion, we report here that TA patients had higher serum sdLDL‐C and Lp(a) concentrations than healthy individuals. Also, TA patients with coronary arteriostenosis had significantly higher serum levels of sdLDL‐C and Lp(a) than those without coronary arteriostenosis. Additionally, serum sdLDL‐C and Lp(a) concentrations and the sdLDL‐C/LDL‐C ratio might be used as diagnostic factors for coronary arteriostenosis in TA patients.

## CONFLICT OF INTEREST

The authors declare that there is no conflict of interest.

## Data Availability

All data are available from the corresponding author upon reasonable request.

## References

[1] Subramanyan R , Joy J , Balakrishnan KG . Natural history of aortoarteritis (Takayasu's disease). Circulation. 1989;80(3):429‐437. 2569946 10.1161/01.cir.80.3.429

[2] Johnston SL , Lock RJ , Gompels MM . Takayasu arteritis: a review. J Clin Pathol. 2002;55(7):481‐486. 12101189 10.1136/jcp.55.7.481 PMC1769710

[3] Frovig AG , Loken AC . The syndrome of obliteration of the arterial branches of the aortic arch, due to arteritis; a post‐mortem angiographic and pathological study. Acta Psychiatr Neurol Scand. 1951;26(3–4):313‐337. 14933169

[4] Rav‐Acha M , Plot L , Peled N , et al. Coronary involvement in Takayasu's arteritis. Autoimmun Rev. 2007;6(8):566‐571. 17854750 10.1016/j.autrev.2007.04.001

[5] Endo M , Tomizawa Y , Nishida H , et al. Angiographic findings and surgical treatments of coronary artery involvement in Takayasu arteritis. J Thorac Cardiovasc Surg. 2003;125(3):570‐577. 12658199 10.1067/mtc.2003.39

[6] Yang L , Zhang H , Jiang X , et al. Clinical manifestations and longterm outcome for patients with Takayasu arteritis in China. J Rheumatol. 2014;41(12):2439‐2446. 25274886 10.3899/jrheum.140664

[7] Lee GY , Jang SY , Ko SM , et al. Cardiovascular manifestations of Takayasu arteritis and their relationship to the disease activity: analysis of 204 Korean patients at a single center. Int J Cardiol. 2012;159(1):14‐20. 21354639 10.1016/j.ijcard.2011.01.094

[8] Lei C , Huang Y , Yuan S , et al. Takayasu arteritis with coronary artery involvement: differences between pediatric and adult patients. Can J Cardiol. 2020;36(4):535‐542. 31924450 10.1016/j.cjca.2019.08.039

[9] Li J , Li H , Sun F , et al. Clinical characteristics of heart involvement in Chinese patients with takayasu arteritis. J Rheumatol. 2017;44(12):1867‐1874. 28811356 10.3899/jrheum.161514

[10] Verma H , Baliga K , George RK , et al. Surgical and endovascular treatment of occlusive aortic syndromes. J Cardiovasc Surg (Torino). 2013;54(1 Suppl 1):55‐69. 23443590

[11] Kuijer A , van Oosterhout MF , Kloppenburg GT , et al. Coronary artery bypass grafting in Takayasu’s disease – importance of the proximal anastomosis: a case report. J Med Case Rep. 2015;9:283. 26666882 10.1186/s13256-015-0767-5 PMC4678760

[12] Sun T , Zhang H , Ma W , et al. Coronary artery involvement in takayasu arteritis in 45 Chinese patients. J Rheumatol. 2013;40(4):493‐497. 23418378 10.3899/jrheum.120813

[13] Park MC , Lee SW , Park YB , et al. Clinical characteristics and outcomes of Takayasu's arteritis: analysis of 108 patients using standardized criteria for diagnosis, activity assessment, and angiographic classification. Scand J Rheumatol. 2005;34(4):284‐292. 16195161 10.1080/03009740510026526

[14] Hlavaty L , Diaz F , Sung L . Takayasu arteritis of the coronary arteries presenting as sudden death in a white teenager. Am J Forensic Med Pathol. 2015;36(3):221‐223. 26110486 10.1097/PAF.0000000000000179

[15] Spagnolo EV , Cannavo G , Mondello C , et al. Unexpected death for Takayasu aortitis associated with coronary ostial stenosis: case report. Am J Forensic Med Pathol. 2015;36(2):88‐90. 25899030 10.1097/PAF.0000000000000154

[16] Kang EJ , Kim SM , Choe YH , et al. Takayasu arteritis: assessment of coronary arterial abnormalities with 128‐section dual‐source CT angiography of the coronary arteries and aorta. Radiology. 2014;270(1):74‐81. 24009351 10.1148/radiol.13122195

[17] Wang X , Chen B , Lv N , et al. Association of abnormal lipid spectrum with the disease activity of Takayasu arteritis. Clin Rheumatol. 2015;34(7):1243‐1248. 25388645 10.1007/s10067-014-2819-4

[18] Alibaz‐Oner F , Koster MJ , Unal AU , et al. Assessment of the frequency of cardiovascular risk factors in patients with Takayasu's arteritis. Rheumatology (Oxford). 2017;56(11):1939‐1944. 28968808 10.1093/rheumatology/kex300

[19] Guleria A , Misra DP , Rawat A , et al. NMR‐based serum metabolomics discriminates takayasu arteritis from healthy individuals: a proof‐of‐principle study. J Proteome Res. 2015;14(8):3372‐3381. 26081138 10.1021/acs.jproteome.5b00422

[20] Feng Y , Tang X , Liu M , et al. Clinical study of children with Takayasu arteritis: a retrospective study from a single center in China. Pediatr Rheumatol Online J. 2017;15(1):29. 28416004 10.1186/s12969-017-0164-2 PMC5393038

[21] Wang X , Dang A , Lv N , et al. High‐sensitivity C‐reactive protein predicts adverse cardiovascular events in patients with Takayasu arteritis with coronary artery involvement. Clin Rheumatol. 2016;35(3):679‐684. 25665822 10.1007/s10067-015-2873-6

[22] de Carvalho JF , Bonfa E , Bezerra MC , et al. High frequency of lipoprotein risk levels for cardiovascular disease in Takayasu arteritis. Clin Rheumatol. 2009;28(7):801‐805. 19283329 10.1007/s10067-009-1153-8

[23] de Graaf J , Hak‐Lemmers HL , Hectors MP , et al. Enhanced susceptibility to in vitro oxidation of the dense low density lipoprotein subfraction in healthy subjects. Arterioscler Thromb. 1991;11(2):298‐306. 1998647 10.1161/01.atv.11.2.298

[24] Chancharme L , Therond P , Nigon F , et al. Cholesteryl ester hydroperoxide lability is a key feature of the oxidative susceptibility of small, dense LDL. Arterioscler Thromb Vasc Biol. 1999;19(3):810‐820. 10073990 10.1161/01.atv.19.3.810

[25] Shen H , Xu L , Lu J , et al. Correlation between small dense low‐density lipoprotein cholesterol and carotid artery intima‐media thickness in a healthy Chinese population. Lipids Health Dis. 2015;14(1):137. 26510458 10.1186/s12944-015-0143-x PMC4625741

[26] Qi Y , Liu J , Wang W , et al. High sdLDL cholesterol can be used to reclassify individuals with low cardiovascular risk for early intervention: findings from the chinese multi‐provincial cohort study. J Atheroscler Thromb. 2020;27(7):695‐710. 31666437 10.5551/jat.49841 PMC7406409

[27] Wang S , Wang X , Zhao Y , et al. Characterizing lipid profiles associated with asymptomatic intracranial arterial stenosis in rural‐dwelling adults: a population‐based study. J Clin Lipidol. 2020;14(3):371‐380. 32389549 10.1016/j.jacl.2020.04.005

[28] Wang FH , Liu J , Deng QJ , et al. Association between plasma essential amino acids and atherogenic lipid profile in a Chinese population: a cross‐sectional study. Atherosclerosis. 2019;286:7‐13. 31071661 10.1016/j.atherosclerosis.2019.04.225

[29] Gaubatz JW , Heideman C , Gotto AJ , et al. Human plasma lipoprotein [a]. Structural properties. J Biol Chem. 1983;258(7):4582‐4589. 6220008

[30] Dahlen GH , Guyton JR , Attar M , et al. Association of levels of lipoprotein Lp(a), plasma lipids, and other lipoproteins with coronary artery disease documented by angiography. Circulation. 1986;74(4):758‐765. 2944670 10.1161/01.cir.74.4.758

[31] Alarcon GJ , Rodriguez SS , Muniz GO , et al. Vascular lesions in patient with Takayasu arteritis and massive elevated lipoprotein(a) levels. Residual involvement or premature aterosclerosis? Clin Investig Arterioscler. 2017;29(2):98‐102. 10.1016/j.arteri.2016.10.002 28188021

[32] Arend WP , Michel BA , Bloch DA , et al. The American College of Rheumatology 1990 criteria for the classification of Takayasu arteritis. Arthritis Rheum. 1990;33(8):1129‐1134. 1975175 10.1002/art.1780330811

[33] Ueno A , Awane Y , Wakabayashi A , et al. Successfully operated obliterative brachiocephalic arteritis (Takayasu) associated with the elongated coarctation. Jpn Heart J. 1967;8(5):538‐544. 5299711 10.1536/ihj.8.538

[34] Lupi E , Sanchez G , Horwitz S , et al. Pulmonary artery involvement in Takayasu's arteritis. Chest. 1975;67(1):69‐74. 14812 10.1378/chest.67.1.69

[35] Kerr GS , Hallahan CW , Giordano J , et al. Takayasu arteritis. Ann Intern Med. 1994;120(11):919‐929. 7909656 10.7326/0003-4819-120-11-199406010-00004

[36] Xuesong F , Enshi W , Jianxun H , et al. Comparison of seven different reagents of peroxidase method for small and dense low density lipoprotein cholesterol (sdLDL‐C) measurement. J Clin Lab Anal. 2021;35(3):e23660. 33377258 10.1002/jcla.23660 PMC7957989

[37] Matsubara O , Kuwata T , Nemoto T , et al. Coronary artery lesions in Takayasu arteritis: pathological considerations. Heart Vessels. 1992;7(S1):26‐31 10.1007/BF01744540 1360966

[38] Seyahi E , Ugurlu S , Cumali R , et al. Atherosclerosis in Takayasu arteritis. Ann Rheum Dis. 2006;65(9):1202‐1207. 16439439 10.1136/ard.2005.047498 PMC1798281

[39] Cipriano PR , Silverman JF , Perlroth MG , et al. Coronary arterial narrowing in Takayasu's aortitis. Am J Cardiol. 1977;39(5):744‐750. 16478 10.1016/s0002-9149(77)80139-2

[40] Wang X , Dang A , Lv N , et al. Long‐term outcomes of coronary artery bypass grafting versus percutaneous coronary intervention for Takayasu arteritis patients with coronary artery involvement. Semin Arthritis Rheum. 2017;47(2):247‐252. 28457530 10.1016/j.semarthrit.2017.03.009

[41] Mukhtyar C , Guillevin L , Cid MC , et al. EULAR recommendations for the management of large vessel vasculitis. Ann Rheum Dis. 2009;68(3):318‐323. 18413441 10.1136/ard.2008.088351

[42] O'Connor TE , Carpenter HE , Bidari S , et al. Role of inflammatory markers in Takayasu arteritis disease monitoring. BMC Neurology. 2014;14(1):62. 24678735 10.1186/1471-2377-14-62 PMC4012521

[43] Superko HR , Gadesam RR . Is it LDL particle size or number that correlates with risk for cardiovascular disease? Curr Atheroscler Rep. 2008;10(5):377‐385. 18706278 10.1007/s11883-008-0059-2

[44] Kulanuwat S , Tungtrongchitr R , Billington D , et al. Prevalence of plasma small dense LDL is increased in obesity in a Thai population. Lipids Health Dis. 2015;14(1):30. 25925050 10.1186/s12944-015-0034-1 PMC4415445

[45] Hoogeveen RC , Gaubatz JW , Sun W , et al. Small dense low‐density lipoprotein‐cholesterol concentrations predict risk for coronary heart disease: the Atherosclerosis Risk In Communities (ARIC) study. Arterioscler Thromb Vasc Biol. 2014;34(5):1069‐1077. 24558110 10.1161/ATVBAHA.114.303284 PMC3999643

[46] Fernandez‐Cidon B , Candas‐Estebanez B , Ribalta J , et al. Precipitated sdLDL: an easy method to estimate LDL particle size. J Clin Lab Anal. 2020;34(7):e23282. 32198796 10.1002/jcla.23282 PMC7370712

[47] Nakano S , Kuboki K , Matsumoto T , et al. Small, dense LDL and high‐sensitivity C‐reactive protein (hs‐CRP) in metabolic syndrome with type 2 diabetes mellitus. J Atheroscler Thromb. 2010;17(4):410‐415. 20197633 10.5551/jat.1891

[48] Kathiresan S , Otvos JD , Sullivan LM , et al. Increased small low‐density lipoprotein particle number: a prominent feature of the metabolic syndrome in the Framingham Heart Study. Circulation. 2006;113(1):20‐29. 16380547 10.1161/CIRCULATIONAHA.105.567107

[49] Manabe Y , Morihara R , Matsuzono K , et al. Estimation of the presence of small dense lipoprotein cholesterol in acute ischemic stroke. Neurol Int. 2015;7(1):5973. 26294946 10.4081/ni.2015.5973 PMC4508545

[50] Sugino I , Kuboki K , Matsumoto T , et al. Influence of fatty liver on plasma small, dense LDL‐ cholesterol in subjects with and without metabolic syndrome. J Atheroscler Thromb. 2011;18(1):1‐7. 21041984 10.5551/jat.5447

[51] Kikkawa K , Nakajima K , Shimomura Y , et al. Small dense LDL cholesterol measured by homogeneous assay in Japanese healthy controls, metabolic syndrome and diabetes patients with or without a fatty liver. Clin Chim Acta. 2015;438:70‐79. 25050800 10.1016/j.cca.2014.07.017

[52] Gerber PA , Thalhammer C , Schmied C , et al. Small, dense LDL particles predict changes in intima media thickness and insulin resistance in men with type 2 diabetes and prediabetes–a prospective cohort study. PLoS One. 2013;8(8):e72763. 23951331 10.1371/journal.pone.0072763 PMC3738563

[53] Nishikura T , Koba S , Yokota Y , et al. Elevated small dense low‐density lipoprotein cholesterol as a predictor for future cardiovascular events in patients with stable coronary artery disease. J Atheroscler Thromb. 2014;21(8):755‐767. 24717762 10.5551/jat.23465

[54] Huang YC , Chang PY , Hwang JS , et al. Association of small dense lowdensity lipoprotein cholesterol in type 2 diabetics with coronary artery disease. Biomed J. 2014;37(6):375‐379. 25179702 10.4103/2319-4170.132883

[55] Taleb A , Witztum JL , Tsimikas S . Oxidized phospholipids on apoB‐100‐containing lipoproteins: a biomarker predicting cardiovascular disease and cardiovascular events. Biomark Med. 2011;5(5):673‐694. 22003918 10.2217/bmm.11.60 PMC3230643

[56] Pearson K , Rodriguez F . Lipoprotein(a) and cardiovascular disease prevention across diverse populations. Cardiol Ther. 2020. 10.1007/s40119-020-00177-4 PMC7584702 32451810

[57] Topciu SV , Begolli L , Kryeziu E . Lipoprotein (a) as an acute phase reactant in patients on chronic hemodialysis. Bosn J Basic Med Sci. 2010;10(1):19‐25. 20192926 10.17305/bjbms.2010.2728 PMC5596605

[58] Noma A , Abe A , Maeda S , et al. Lp(a): an acute‐phase reactant? Chemist Phys Lipids. 1994;67‐68:411‐417. 10.1016/0009-3084(94)90164-3 7514505

[59] Cas MD , Roda G , Li F , et al. Functional lipids in autoimmune inflammatory diseases. Int J Mol Sci. 2020;21(9):3074. 10.3390/ijms21093074 PMC7246500 32349258

[60] Roldan PC , Greene ER , Qualls CR , et al. Progression of atherosclerosis versus arterial stiffness with age within and between arteries in systemic lupus erythematosus. Rheumatol Int. 2019;39(6):1027‐1036. 30877372 10.1007/s00296-019-04267-y

[61] Benagiano M , Borghi MO , Romagnoli J , et al. Interleukin‐17/Interleukin‐21 and Interferon‐gamma producing T cells specific for beta2 Glycoprotein I in atherosclerosis inflammation of systemic lupus erythematosus patients with antiphospholipid syndrome. Haematologica. 2019;104(12):2519‐2527. 30872365 10.3324/haematol.2018.209536 PMC6959190

[62] Atta AM , Silva J , Santiago MB , et al. Clinical and laboratory aspects of dyslipidemia in Brazilian women with systemic lupus erythematosus. Clin Rheumatol. 2018;37(6):1539‐1546. 29516281 10.1007/s10067-018-4051-0

